# Supplementary material for: The Spectrum and Carrier Frequencies of Common Pathogenic Cystic Fibrosis Transmembrane Conductance Regulator Gene Mutations in Men from the General Population: The Role of Ethnicity
Source: Int J Mol Sci. 2025 Jul 10;26(14):6625. doi: 10.3390/ijms26146625 (PMC12294213; doi:10.3390/ijms26146625)
Supplement: Supplementary file 1 [file ijms-26-06625-s001.zip › ijms-3683063-supplementary.pdf]

Supplementary Table S1. Spectrum and carrier frequencies of pathogenic CFTR gene mutations in Slavic men from Russia and Belarus (carriers' number/carrier frequency/95% CI)

**CFTR genotype**	**Russian Slavs, n=704**	**Belarusian Slavs, n=482**	**Total Slavs, n=1186**
F508del/N	14/0.0199 (0.0096-0.0302)	10/0.0207 (0.0080-0.0335)	24/0.0202 (0.0122-0.0282)
G542X/N	0/0.0000 (0.0000-0.0000)	0/0.0000 (0.0000-0.0000)	0/0.0000 (0.0000-0.0000)
N1303K/N	1/0.0014 (0.0000-0.0042)	1/0.0021 (0.0000-0.0061)	2/0.0017 (0.0000-0.0040)
3849+10kbC>T/N	0/0.0000 (0.0000-0.0000)	1/0.0021 (0.0000-0.0061)	1/0.0008 (0.0000-0.0025)
CFTRdele2,3/N	1/0.0014 (0.0000-0.0042)	0/0.0000 (0.0000-0.0000)	1/0.0008 (0.0000-0.0025)
R117C/N	1/0.0014 (0.0000-0.0042)	0/0.0000 (0.0000-0.0000)	1/0.0008 (0.0000-0.0025)
F508del/5T	1/0.0014 (0.0000-0.0042)	0/0.0000 (0.0000-0.0000)	1/0.0008 (0.0000-0.0025)
F508del/5T-12TG	0/0.0000 (0.0000-0.0000)	1/0.0021 (0.0000-0.0061)	1/0.0008 (0.0000-0.0025)
R117C/5T	0/0.0000 (0.0000-0.0000)	1/0.0021 (0.0000-0.0061)	1/0.0008 (0.0000-0.0025)
Total heterozygotes	**18/0.0256 (0.0139-0.0372)**	**14/0.0290 (0.0141-0.0440)**	**32/0.0270 (0.0178-0.0362)**
IVS9-5T/N	61/0.0866 (0.0659-0.1074)	29/0.0602 (0.0389-0.0814)	90/0.0759 (0.0608-0.0910)
5T/5T	0/0.0000 (0.0000-0.0000)	1/0.0021 (0.0000-0.0061)	1/0.0008 (0.0000-0.0025)
5T-12TG/N	3/0.0043 (0.0000-0.0091)	5/0.0104 (0.0013-0.0194)	8/0.0067 (0.0021-0.0114)
5T-13TG/N	0/0.0000 (0.0000-0.0000)	0/0.0000 (0.0000-0.0000)	0/0.0000 (0.0000-0.0000)
Total polymorphisms	**64/0.0909 (0.0697-0.1121)**	**35/0.0726 (0.0494-0.0958)**	**99/0.0835 (0.0677-0.0992)**

No significant differences in the total carrier frequency of pathogenic CFTR variants (χ^2=0.67; p=0.413) as well as in the total carrier frequency of heterozygotes (χ^2=0.14; p=0.707) or in the total carrier frequency of polymorphisms (χ^2=1.300; p=0.254) were found between the Russian and Belorussian Slavs. Additionally, there were also no significant differences between Russian and Belarusian Slavs in the carrier frequency of IVS9-5T polymorphism (χ^2=1.30; p = 0.087). The carrier frequencies of pathogenic CFTR variants were compared by the Yate's Chi-squared (χ^2) test.

Supplementary Table S2. Geographic distribution of the studied population

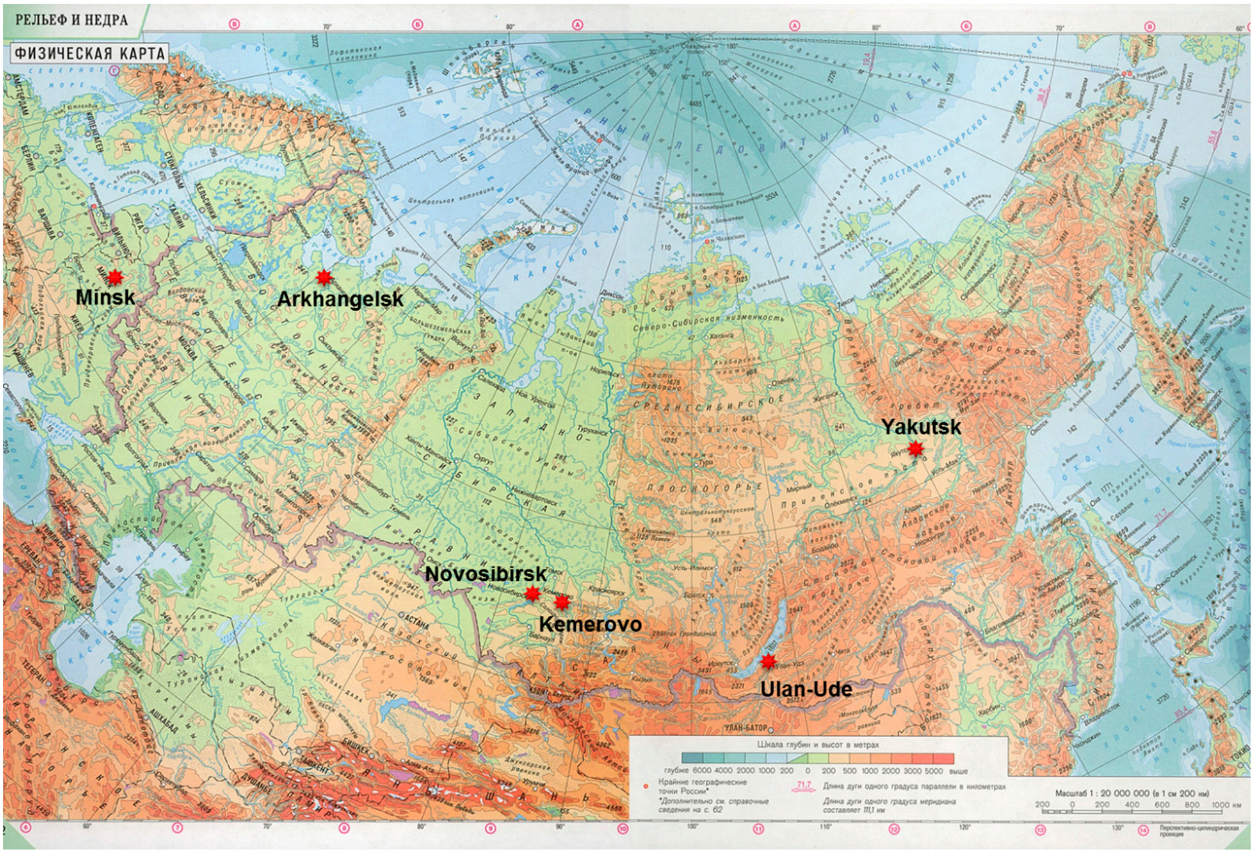

Six cities selected for this study located in the Russian Federation (Arkhangelsk, Novosibirsk, Kemerovo, Ulan-Ude and Yakutsk) and Belorussia (Minsk), where three large ethnic populations (Slavs, Buryats and Yakuts) live compactly. The cities where the population we are studying lives are marked on the map with asterisks.

Supplementary Table S3. Medical and genetic information on the CFTR gene mutations and polymorphisms analyzed in the current study

N	Variant legacy name	Variant protein name	Class	Phenotype	Clinical significance	%
1	F508del	p.(Phe508del)	II	severe	CF-causing	51.4
2	CFTRdele2,3	p.(Ser18Argfs*16)	I	severe	CF-causing	6.10
3	E92K	p.(Glu92Lys)	II	mild	CF-causing	3.71
4	1677delTA	p.(Tyr515*)	I	severe	CF-causing	2.51
5	3849+10kbC->T	No protein name	V	mild	CF-causing	2.24
6	2143delT	p.(Leu671*)	I	severe	CF-causing	1.99
7	2184insA	p.(Gln685Thrfs*4)	I	severe	CF-causing	1.93
8	W1282X	p.(Trp1282*)	I	severe	CF-causing	1.73
9	L138ins	p.(Leu138dup)	IV	mild	CF-causing	1.71
10	N1303K	p.(Asn1303Lys)	II	severe	CF-causing	1.62
11	G542X	p.(Gly542*)	I	severe	CF-causing	1.49
12	R334W	p.(Arg334Trp)	IV	mild	CF-causing	0.75
13	S1196X	p.(Ser1196*)	I	severe	CF-causing	0.45
14	I507del[+]	p.(Ile507del)	II	severe	CF-causing	0.45*
15	R117C	p.(Arg117Cys)	V	mild	CF-causing	0.10
16	E92X	p.(Glu92*)	I	severe	CF-causing	0.03
17	L467F/F508del	p.(Leu467Phe/Phe508del)	A complex allele L467F/F508del leads to ineffectiveness of therapy			0.87
19	IVS8-5T	No protein name	V	mild	Varying clinical consequence	0.01
20	5T-12TG	No protein name	V	mild	Varying clinical consequence	0.03
21	5T-13TG[+]	No protein name	V	mild	Varying clinical consequence	0.06*

Note: information taken from [14; 17]; % - the allele frequency of pathogenic variant [14]; [+] - the genetic variant is not registered in “The Register of Patients with Cystic Fibrosis in the Russian Federation. 2023 Year” [14]; * - the value was taken from “The CFTR2 base” [17].

Supplementary Table S4. Sequences of oligonucleotide primers for the studied regions of the CFTR gene

**CFTR variant**	**Oligonucleotide primers 5`- 3`**	
	direct	reverse
G542X	CTACCAAATCTGGATACTATACC	GCAATAGTGTGATATATGATTAC
F508del, I507del	GATCCATTCACAGTAGCTTACCCA	CAAGTGAATCCTGAGCGTGAT
E92K, E92X	GTGCTAAGAGTTTCACATATGGTATG	GAGCGTTCCTCCTTGTTATCC
L138ins	CGCGATTTATCTAGGCATAGGC	CTTACTTGTACCAGCTCACTACC
W1282X	GGCATGGTACCTATATGTCACAG	GCCTATGAGAAAACTGCACTGG
N1303K	GTCAGCTATATCAGCCATTTGTGT	GAGAGAACTTGATGGTAAGTACATG
2184insA	TGCATGAAGGTAGCAGCTATT	GAATCCTCTTCGATGCCATTCA
3849+10kb C>T	GACTTGTCATCTTGATTTCTGGAG	TAACTTCAATGCACCTCCTCC
2143delT	TTGCATGAAGGTAGCAGCTAT	CCTCTTCGATGCCATTCATTTG
1677delTA	CACTTCTGCTTAGGATGATAATTGG	GACTAACCGATTGAATATGGAGC
R334W	GAACAGAACTGAAACTGACTCGGAA	GCTCCAAGAGAGTCATACCATGT
S1196X	GATCTGTGAGCCGAGTCTTTA	CACATTGCTTCAGGCTACTGG
CFTRdele2,3 (21kb)	TGGTGTTTACCTACCTAGAGAAAG	CACTCAGAACCCATCATAGGATAC
5/7/9T, (TG)n	GGCCATGTGCTTTTCAAACTAA	GAGGCTGTCATCACCATTAGAAG
Exon 3	GCACATGCAACTTATTGGTCC	CACCTATTCACCAGATTTCGTAGTC
R117C	GTGCTAAGAGTTTCACATATGGTATG	GAGCGTTCCTCCTTGTTATCC
L467F	GATCCATTCACAGTAGCTTACCCA	CAAGTGAATCCTGAGCGTGAT

For validation we used sequencing of the studied CFTR gene regions containing mutations. We attach tables with primers and probes that we used in our mutation detection method (Supplementary Table S5). All probes are designed in such a way that they are completely complementary to the sequences containing the mutant allele of CFTR. In the case of complete complementarity with mutant allele, we observe a peak at high temperature in PCR, and in the case of a normal allele, a peak at low temperature (Supplementary Table 6). The method was validated using Sanger sequencing (Supplementary Table S6).

Supplementary Table S5. Oligonucleotides for mutation analysis of the CFTR gene

CFTR variant	Primer	Sequence 5`-3`
G542X	542-F	CAGCAAATGCTTGCTAGACCAAT
	542-R	CCAAGTTTGCAGAGAAAGACAA(T-BHQ1)AT
	542-P	CCTTCTCAAAGAACTA(FAM)
F508del/507del	508-F	GTAGACTAACCGATTGAATATGGAGC
	508-R	TATGCCTGGCACCAT(T-BHQ2)AAAGA
	508-P	GGAAACACCAATGITATTT(ROX)
	507d-P	GGAAACACCAAAGITATTT(ROX)
L467F	467-F	CCTCTGAAGGCTCCAGTTCT
	467-R	CCTAATAATGATGGGTTTTATT(T-BHQ2)CC
	467-P	CATTAGAAATGAAGTC(ROX)
E92K/E92X	92-F	GTCTTGTGTTGAAATTCTCAGGGT
	92-R	CAGTAAGAGAGGCTGTAC(T-BHQ2)GCTT
	92K-P	CCTTTTGTAGAAAGTCAC(ROX)
	92X-P	CCCCTTTTGTAGTAAGTCAC(HEX)
R117C	117-F	GCCTCTCTTACTGGGAAGAAT
	117-R	CATAAGCCTATGCCTAGATAAA(T-BHQ1)CG
	117C-P	GAGGAATGCTCTATC(HEX)
L138ins Leu	138-F	TTCTCATCTGCATTCCAATGTGAT
	138-R	CTCTTTATTGTGAGGACAC(T-BHQ1)GC
	138-P	TGGTTGGGTGTAGTAGGAGC(FAM)
W1282X	1282-F	CCAGATCGATGGTGTGTCTT
	1282-R	CTCACCTGTGGTATCAC(T-BHQ1)CCAA
	1282X-P	CAACAGTGAAGGAAAGC(HEX)
N1303K	1303-F	CAGTTAGCAGCCTTACCTCATCT
	1303-R	GAAAGTATTTATTTTTTCTGGAACA(T-BHQ2)TTAG
	1303-P	GGGATCCAACTTTTTTCT(ROX)
2184insA	685-F	CAATCCTAACTGAGACCTTACACC
	685-R	CCCAAACTCTCCAGTCTG(T-BHQ1)TTA
	685-P	GACAGAAACAAAAAAAACAATC(HEX)
3849+10kbC/T	3849-F	CTAGCTGTAATTGCATTGTACCATGA
	3849-R	GAGTCTTCCATCTGT(T-BHQ2)GCAGTA
	3849-P	GTCTTACTCACCATTTT(ROX)
2143delT	671-F	CAAACTCTCCAGTCTGTTTAAAAGATTG
	671-R	CAATCCTAACTGAGACCT(T-BHQ1)ACAC
	671-P	CCTTCTATGAGAAACGGT(FAM)
1677delTA	515-F	GCACAGTGGAAGAATTTCATTCTG
	515-R	CTCTTCTAGTTGGTATGCTT(T-BHQ1)GATG
	515-P	GATGAATAGATACAGAAGCGT(HEX)
R334W	334-F	GCAGAACAATGCAGAATGAGATG
	334-R	GCTTCCCTATGCACTAA(T-BHQ1)CAAAG
	334-P	ATTTTCCAGAGGATGATTC(FAM)

S1196X	1196-F	GCTGTGAGATCTTTGACAGTCATTT
	1196-R	GCCAACTCTCGAAAGTTATGA(T-BHQ2)TAT
	1196-P	CTTCACGTGTCAATTCTC(ROX)
CFTRdele2,3	Del21-F	TGGTGTTTACCTACCTAGAGAAAG
	Del21-R	CACTCAGAACCCATCATAGGATAC
	Del21-P	(FAM)CACAAGGC(T-BHQ1)TGTCTTTTACCCTGCCAAAAGCp
5/7/9T (IVS8-5T)	IVS8-F	GGCCATGTGCTTTTCAAACTAA
	IVS8-R	GTTTTGCTTTCTCAAATAAT(T-BHQ1)CCCC
	5T	GTGTTTTTAACAGGGATTT(ROX)
	7T	GTGTTTTTTTAACAGGGATTT(HEX)
	9T	GTGTTTTTTTTTAACAGGGATTT(FAM)
(TG)n (12/13TG)	8-F	GGCCATGTGCTTTTCAAACTAA
	8-R	GAGGCTGTCATCACCATTAGAAG
	8-12TG	(ROX)TTGATGTG(T-BHQ2)GTGTGTGTGTGTGTGTGTGTTTTp
	8-13TG	(HEX)TGATGTG(T-BHQ1)GTGTGTGTGTGTGTGTGTGTGTTTTp
Exon 3 CFTR	Ex3-F	GAATGGGATAGAGAGCTGGC
	Ex3-R	TTGATTCCATAGAACATAAATCTCCAG
	Ex3-P	(HEX)TCCTAAAC(T-BHQ1)CATTAATGCCCTTCGGCGATGp

Supplementary Table S6. The results of PCR and sequencing of the studied gene regions in the CFTR gene

CFTR variant	PCR	Sequence
G542X	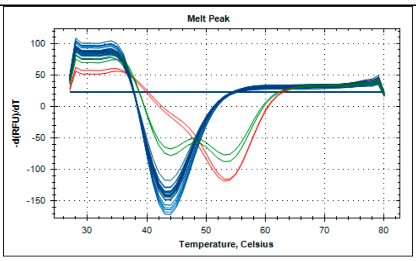<br>Tm=43 – wild type (wt)<br>Tm=53 – mutant type (mut)	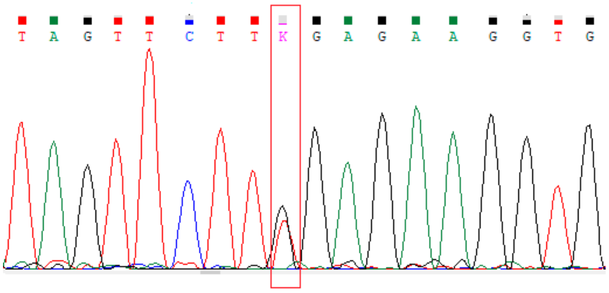<br>c.1624 G>T
F508del	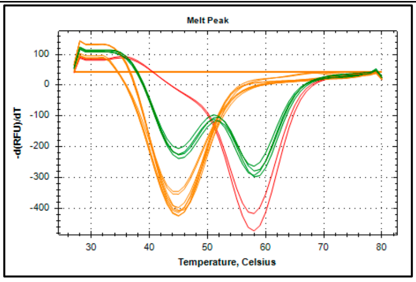<br>Tm=45 – wt<br>Tm=58 – mut	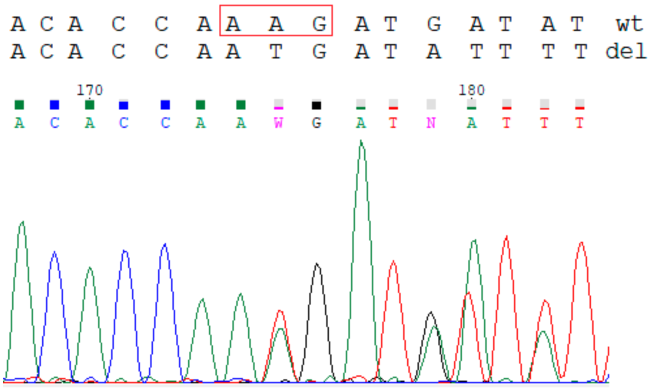
E92K	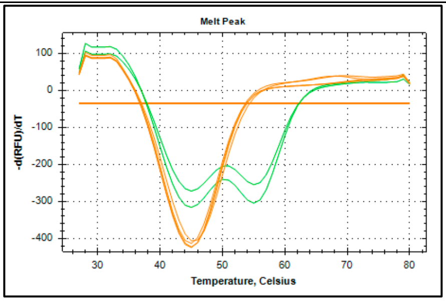<br>Tm=45° - wt<br>Tm=55° - mut	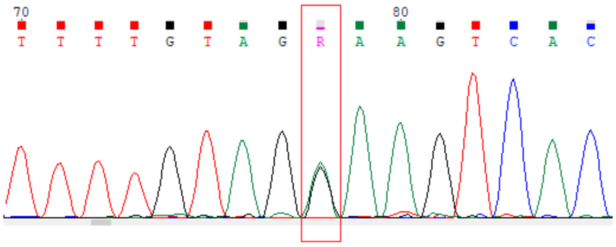<br>c.274 G>A
L138ins Leu	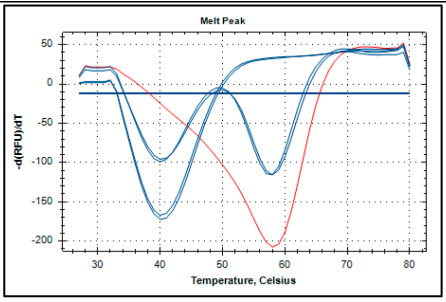<br>Tm=40° - wt<br>Tm=58° - mut	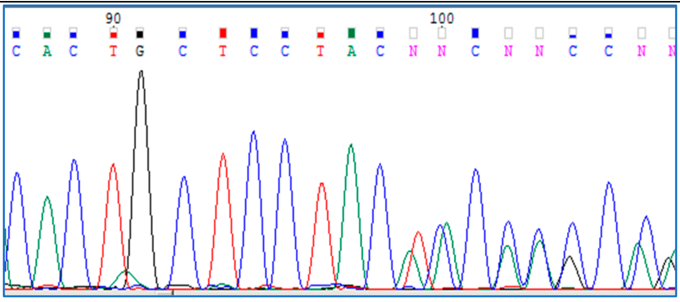<br>c.413-414insTAC
L467F	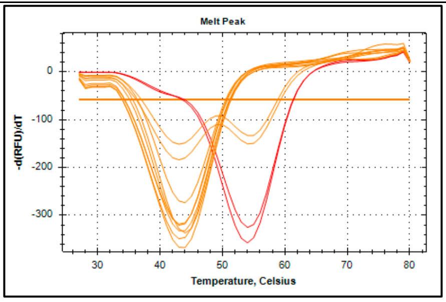<br>Tm=44° - wt	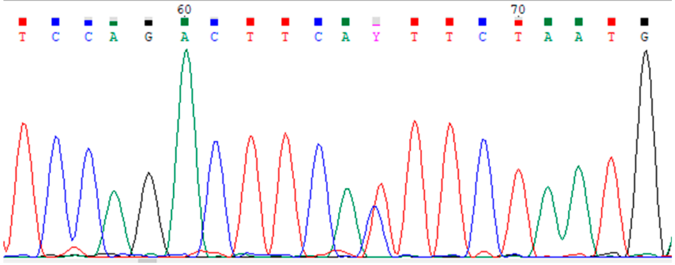<br>c.1399 C>T

	Tm=55° - mut	
N1303K	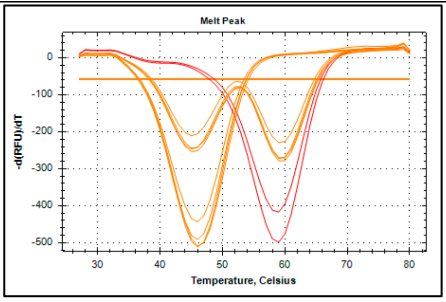<br>Tm=45° - wt<br>Tm=59° - mut	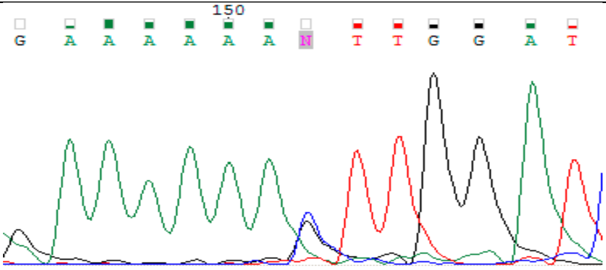<br>c.3909C>G
S1196X	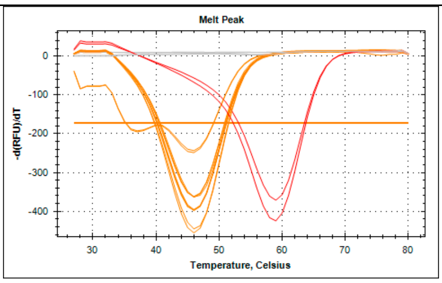<br>Tm=46° - wt<br>Tm=59° - mut<br>Tm=37° - non-targeted mutation	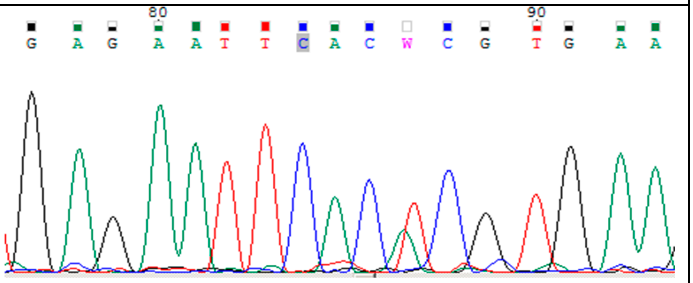<br>c.3590 A/T | p.His1197Leu | H1197L | rs765133036<br>Non-targeted mutation sequence
2143delT	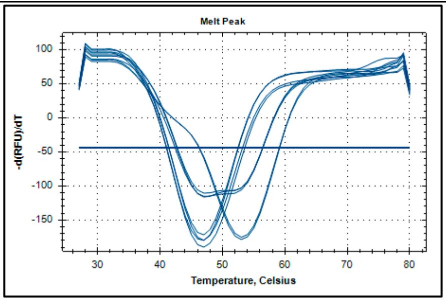<br>Tm=47° - wt<br>Tm=53° - mut	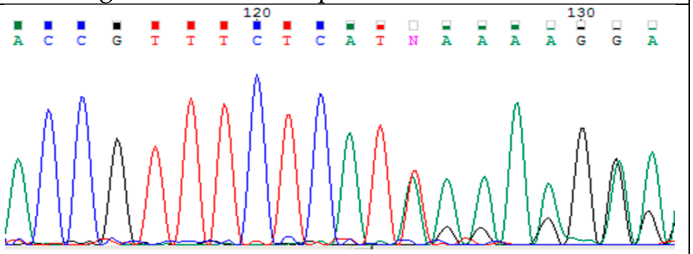<br>c.2143delT
2184insA	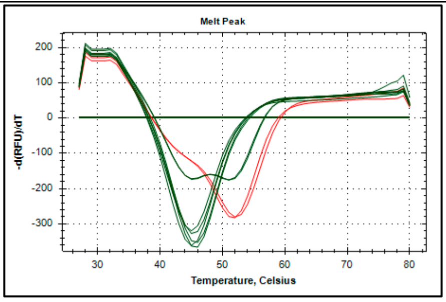<br>Tm=45° - wt<br>Tm=52° - mut	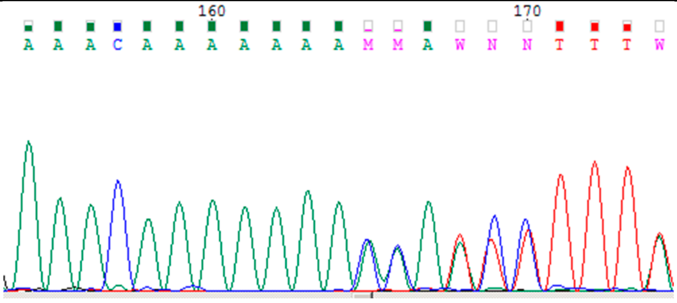<br>c.2184insA
1677delTA	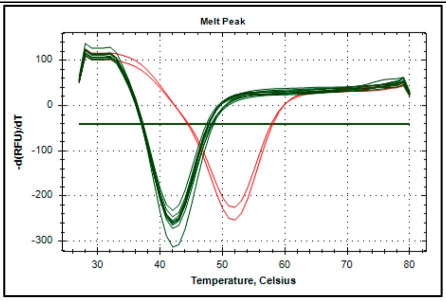<br>Tm=42° - wt<br>Tm=52° - mut	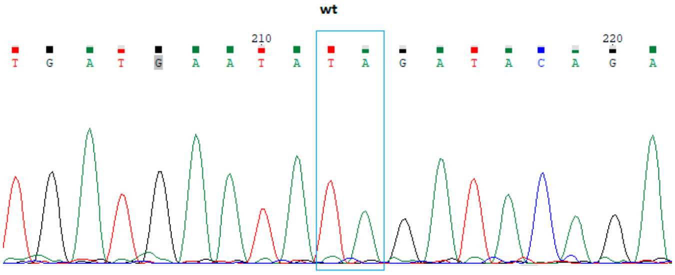<br>c.1677delTA – wild type sequence

<table>
<tr>
<td>R334W</td>
<td>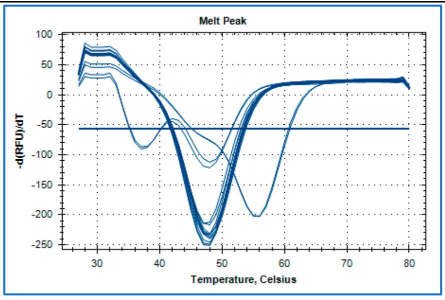

Tm=47° - wt
Tm=55° - mut
Tm=37° - non-targeted mutation</td>
<td>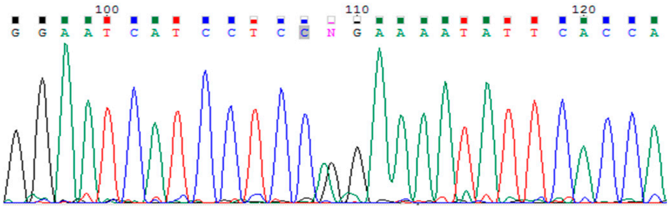
<table><tr><td>c.1001G>A</td><td>p.Arg334 Gln</td><td>R334Q</td><td>rs397508137</td></tr></table>
Non-targeted mutation sequence</td>
</tr>
<tr>
<td>CFTRdele2,3</td>
<td>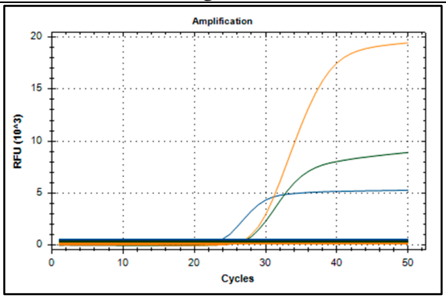

ROX – IC
FAM – del21kb
HEX – exon3 (deletion)</td>
<td>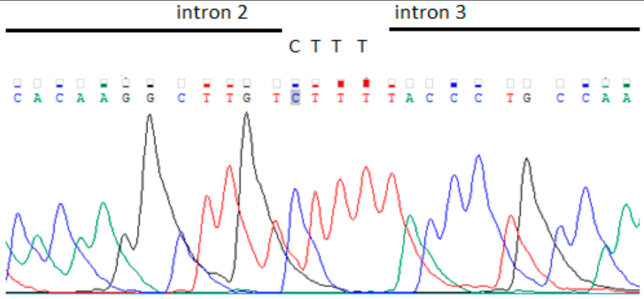

Del/Del</td>
</tr>
<tr>
<td>R117C</td>
<td>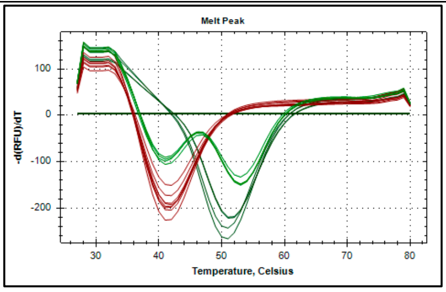

Tm=41 – wt
Tm=51 - mut</td>
<td>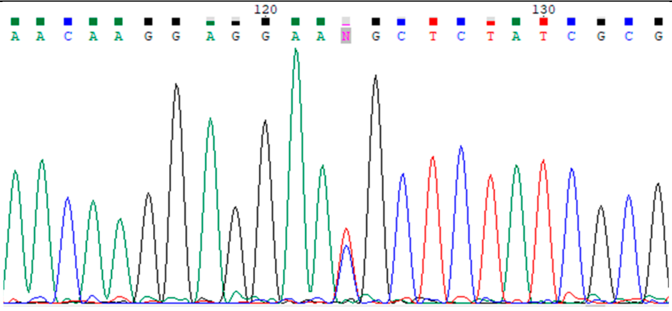
c.349 C>T</td>
</tr>
<tr>
<td>5/7/9T
(IVS8-5T)

12/13TG</td>
<td>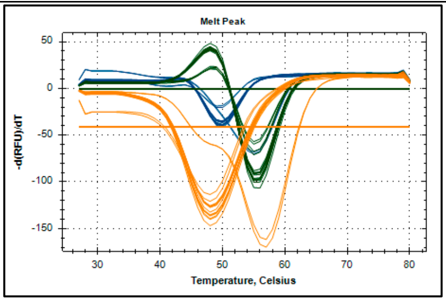

Tm(ROX)=57° - 5T
Tm(HEX)=55° - 7T
Tm(FAM)=55° - 9T
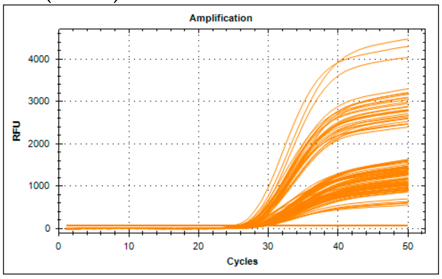

12TG</td>
<td>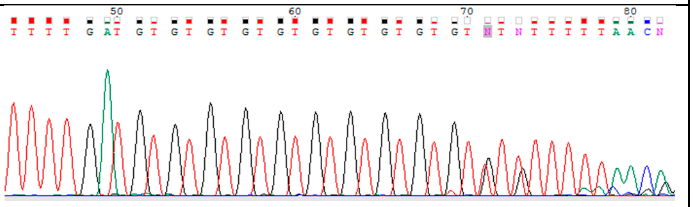
12TG-5T/10TG-9T
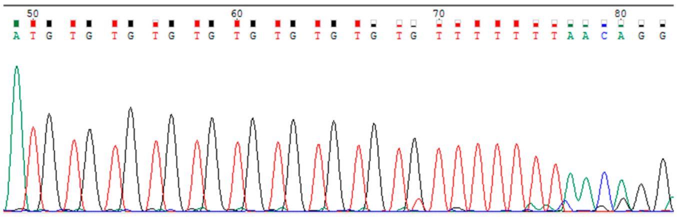
10TG-7T/10TG-7T
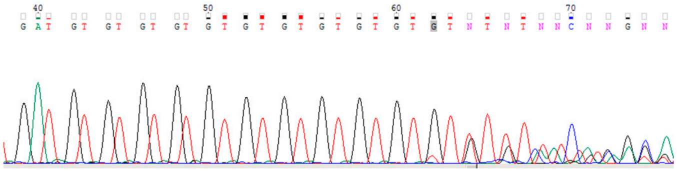
11TG-5T/13TG-5T</td>
</tr>
</table>

	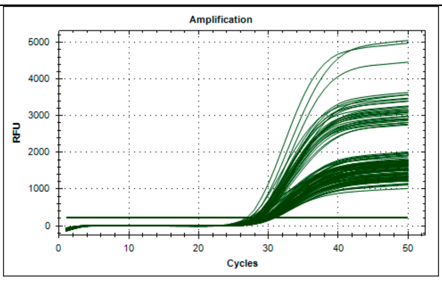Amplification<br>RFU<br>Cycles<br>0<br>1000<br>2000<br>3000<br>4000<br>5000<br>0<br>10<br>20<br>30<br>40<br>50<br>13TG	
